# Cortical microstructural changes predict tau accumulation and episodic memory decline in older adults harboring amyloid

**DOI:** 10.1038/s43856-023-00324-7

**Published:** 2023-08-01

**Authors:** Geoffroy Gagliardi, Elena Rodriguez-Vieitez, Victor Montal, Jorge Sepulcre, Ibai Diez, Cristina Lois, Bernard Hanseeuw, Aaron P. Schultz, Michael J. Properzi, Kathryn V. Papp, Gad A. Marshall, Juan Fortea, Keith A. Johnson, Reisa A. Sperling, Patrizia Vannini

**Affiliations:** 1 Massachusetts General Hospital, Harvard Medical School, Boston, MA 02114 USA; 2 Athinoula A. Martinos Center for Biomedical Imaging, Charlestown, MA 02129 USA; 3 Brigham and Women’s Hospital, Harvard Medical School, Boston, MA 02115 USA; 4 Karolinska Institutet, Department of Neurobiology, Care Sciences and Society, Stockholm, 14152 Sweden; 5 Sant Pau Memory Unit, Department of Neurology, Hospital de la Santa Creu i Sant Pau, Biomedical Research Institute Sant Pau, Universitat Autònoma de Barcelona, Barcelona, 08041 Spain; 6 Centre of Biomedical Investigation Network for Neurodegenerative Diseases (CIBERNED), Madrid, 28031 Spain; 7 Gordon Center for Medical Imaging, Boston, MA 02114 USA; 8 Saint Luc University Hospital, Université Catholique de Louvain, Brussels, 1200 Belgium

**Keywords:** Ageing, Predictive markers, Alzheimer's disease

## Abstract

**Introduction:**

Non-invasive diffusion-weighted imaging (DWI) to assess brain microstructural changes via cortical mean diffusivity (cMD) has been shown to be cross-sectionally associated with tau in cognitively normal older adults, suggesting that it might be an early marker of neuronal injury. Here, we investigated how regional cortical microstructural changes measured by cMD are related to the longitudinal accumulation of regional tau as well as to episodic memory decline in cognitively normal individuals harboring amyloid pathology.

**Methods:**

122 cognitively normal participants from the Harvard Aging Brain Study underwent DWI, T1w-MRI, amyloid and tau PET imaging, and Logical Memory Delayed Recall (LMDR) assessments. We assessed whether the interaction of baseline amyloid status and cMD (in entorhinal and inferior-temporal cortices) was associated with longitudinal regional tau accumulation and with longitudinal LMDR using separate linear mixed-effects models.

**Results:**

We find a significant interaction effect of the amyloid status and baseline cMD in predicting longitudinal tau in the entorhinal cortex (*p* = 0.044) but not the inferior temporal lobe, such that greater baseline cMD values predicts the accumulation of entorhinal tau in amyloid-positive participants. Moreover, we find a significant interaction effect of the amyloid status and baseline cMD in the entorhinal cortex (but not inferior temporal cMD) in predicting longitudinal LMDR (*p* < 0.001), such that baseline entorhinal cMD predicts the episodic memory decline in amyloid-positive participants.

**Conclusions:**

The combination of amyloidosis and elevated cMD in the entorhinal cortex may help identify individuals at short-term risk of tau accumulation and Alzheimer’s Disease-related episodic memory decline, suggesting utility in clinical trials.

## Plain language summary

People with Alzheimer’s disease have problems with their memory and ability to acquire and process knowledge. Understanding the earliest brain changes leading to these problems helps identify those likely to develop Alzheimer’s disease early in the disease process. This study used a marker that measures the mobility of water in the brain to investigate how these changes can predict development of a protein named tau and changes in people’s memory. The participants showed no signs of memory impairment at the beginning of the study, but some developed memory decline during follow-up. Greater mobility of water in certain brain areas predicted future increase in tau and decline in memory, indicating this measure could be used to identify people at risk of developing Alzheimer’s disease.

## Abstract (web summary)

Gagliardi, Rodriguez-Vieitez et al. image brain microstructural changes over time in cognitively-normal older adults. Cortical mean diffusivity was associated with accumulation of tau and episodic memory decline in those with amyloid deposits.

## Introduction

Since the development of various neuroimaging techniques in the last decades, the research on Alzheimer’s disease (AD) has notably focused on the search for early biomarkers. AD has been biologically defined by the presence of both amyloid and tau brain pathology^1–3^. Using several methods, including cerebrospinal fluid (CSF), positron emission tomography (PET), and magnetic resonance imaging (MRI), studies have consistently shown a biological signature that would start to develop years before the clinical diagnosis^1–5^. This accumulation would follow a sequential order, with amyloidosis being the first to aggregate^1^, and has led to the concept of the preclinical stage of AD^1, 3^. Note that not all AD biomarkers become detectable at the same time. With tauopathy, for example, recent studies have shown that while abnormal CSF could be detected years before the diagnosis, PET measures of tau would show changes closer to the diagnosis^6^ with a distribution significantly correlating with clinical symptoms^6,7^.

Combining different techniques, multimodal neuroimaging studies have started to explore the associations and synergies between early AD biomarkers. Unlike PET techniques using radioactive tracers, MRI has the advantage of having no radiation exposure, being more affordable, and being widely available in clinics. Among these MRI techniques, diffusion-weighted imaging (DWI) computes the spatial distribution of the diffusion of water molecules^8^. While DWI is typically quantified in the white matter, recent developments allow exploring AD-related changes on a microstructural scale. Specifically, Montal and colleagues have developed a method to assess microstructural properties in the gray matter by means of the cortical mean diffusivity (cMD)^9,10^. Using this approach, they demonstrated that cognitively healthy older participants with significant amyloidosis in the absence of tau demonstrated a decreased cMD in cross-sectional analyses^9^, a finding that is probably related to amyloid-related inflammation^11^. In contrast, participants with both amyloidosis and tauopathy showed increased cMD values^9^. These results have been replicated using pseudo-longitudinal modeling in autosomal dominant AD looking at individuals across the spectrum of disease as they approach the estimated year of onset of symptoms^10^. The results showed a biphasic trajectory of changes in which preclinical mutation carriers demonstrated first a decrease followed by an increase in cortical diffusivity in preclinical mutation carriers closer to their estimated time of onset^10^. More generally, increased mean diffusivity is observed only in the presence of both amyloidosis and tauopathy^9,12–14^, and may be explained by amyloid-related inflammatory processes^10^. However, since cMD is a relatively recently developed biomarker, more studies are needed to explore the relationships with the pathological AD profile over time.

More recently, in a cross-sectional design examining cognitively normal (CN) older adult participants, Rodriguez-Vieitez and colleagues explored the relationship between cMD and AD pathological hallmarks measured by amyloid and tau PET imaging, and demonstrated that cMD was positively associated with tau but not amyloid burden^15^. In their longitudinal analyses, the authors also found that baseline regional cMD was related to hippocampal atrophy as well as to the progression from CN to mild cognitive impairment (MCI). Similarly, in their pseudo-longitudinal study including familial AD participants, Montal and colleagues also showed a relationship between cMD and the evolution of cortical thickness. Specifically, they found a quadratic relationship between the two variables, starting with an initial increased cortical thickness associated with decreased diffusivity in the absence of detectable tauopathy. As the estimated years to symptoms onset decreases, and tau pathology becomes detectable, the opposite pattern is observed^10^. While these studies have shown an association between microstructural and neurodegenerative changes, as well as with tau cross-sectionally, the association between cMD and subsequent longitudinal accumulation of tau pathology has yet to be explored. Compared to CSF-Tau, tau PET shows tau changes chronologically closer to the diagnosis and is closely associated with clinical symptoms (with a lesional topography relating to the clinical symptomatology)^6,7^. Therefore, predicting future tau PET burden, especially in an at-risk-for AD population, would be clinically meaningful.

To distinguish between asymptomatic at-risk-for AD participants and healthy controls, we stratified our sample into two groups based on the ^11^C-Pittsburgh compound-B (PiB) PET, similar to our previous study^15^. As one of the pathognomonic biomarkers of AD^1–3^, brain amyloidosis accumulates decades ahead of clinical dementia^4,5^ and is part of the NIA-AA Research Framework’s biological definition of AD^1^. Previous studies demonstrated that the topographical pattern of amyloid lesions overlaps with some brain networks, especially with the default mode network (DMN)^16^—a network known to support several cognitive functions such as self-referential processes^17^, episodic memory (EM) or executive functioning^18,19^. These cognitive functions have been identified as showing the earliest subtle cognitive decline in the preclinical stage^20–24^. Recent studies have explored the relationship between cMD and cognitive decline. Fortea and colleagues, focusing on temporal regions (i.e., regions associated with tau accumulation), showed a cross-sectional association between an increased cMD and decreased EM performances^25^. Similarly, Rodriguez-Vieitez and colleagues showed that cMD in the temporal regions was associated with longitudinal cognition using a cognitive composite score^15^. So far, no study has examined this relationship focusing on EM. This information may be clinically important, as episodic memory is one of the earliest cognitive domains to decline in AD^26,27^.

The present work aimed at determining the association between baseline cMD and the longitudinal changes in tau and episodic memory in CN individuals at risk for AD. Here, we show that increased cMD at baseline is associated with a higher rate of tau accumulation and a steeper decline in episodic memory over time. We further demonstrate that amyloid moderates our findings, such that these associations are more pronounced in cognitively normal individuals harboring amyloid pathology.

## Methods

### Population

Participants were selected from the Harvard Aging Brain Study (HABS; https://habs.mgh.harvard.edu), a single-center observational study conducted at the Massachusetts General Hospital. HABS is a longitudinal study focused on older adults, CN at enrollment, aiming to improve our understanding of AD’s preclinical stages. The inclusion criteria for participants to be considered CN at the screening included normal performances (adjusted for age and education) on the Mini-Mental State Examination (MMSE)^28^, Logical Memory (LM)^29^, and Clinical Dementia Rating Scale (CDR = 0)^30^, as well as the absence of significant depression as measured by the Geriatric Depression Scale (GDS < 10/30)^31^. Clinical Status and progression to either MCI or AD dementia were determined in clinical consensus meetings. In addition, all participants underwent brain imaging, including structural MRI (T1-weighted and DWI), as well as PET with amyloid (PiB)^32^ and tau (18F-AV-1451/flortaucipir [FTP])^33^ tracers. Apolipoprotein E4 (*APOE*-ε4) status (presence vs absence of E4 allele), was also collected. The protocol was approved by the Mass General Brigham institutional review board, and study procedures were carried out only after participants reviewed and signed the consent form. The study was carried out following the ethical standards defined in the Declaration of Helsinki (2000).

For the current study, we selected participants with concurrent data on MRI, DWI, FTP-PET, and cognitive assessments. All assessments were performed within one year of the MRI scan. In addition, all participants had at least one longitudinal assessment, including MRI, FTP-PET, and cognition (in addition to the baseline visit). Cognitive assessments were performed in annual visits (i.e., every year). With these criteria, 122 participants were included in the study. Demographic characteristics at baseline are summarized in Table 1.

**Table 1:** Baseline demographics and imaging measures of the study sample and their comparisons between the amyloid groups.

Variables	All	A−	A+	Test statistics	95% CI	*p* values
*N*	122	86	36			
*N* observations	2.4 ± 0.55 [2;4]	2.3 ± 0.5 [2;4]	2.5 ± 0.65 [2;4]	−1.6	−0.42, 0.05	0.12
Age (Years)	71 ± 9.7 [50;90]	69 ± 9.9 [50;90]	76 ± 7.1 [60;89]	−4.5	−10, −4	1.464913e-04*
Sex (F)	79 (64.75%)	58 (67.44%)	21 (58.33%)	0.19	−0.20, 0.58	0.507
Education (years)	16 ± 2.9 [8;20]	16 ± 2.9 [8;20]	16 ± 2.9 [11;20]	0.39	−0.91, 1.4	0.7
APOE (E4 carriers)	37 (30.3%)	15 (17.4%)	22 (61.1%)			4.882349e-06*
Race						0.6
Asian	2 (1.7%)	2 (2.4%)	0 (0%)			
Black	12 (9.9%)	10 (12%)	2 (5.6%)			
Native American	1 (0.8%)	1 (1.2%)	0 (0%)			
N/A	1	1	0			
White	106 (88%)	72 (85%)	34 (94.44%)			
Ethnicity						0.3
Hispanic	4 (3.3%)	4 (4.7%)	0 (0%)			
Non-Hispanic	118 (96.72%)	82 (95.35%)	36 (100%)			
Clinical progression	10 (8.2%)	2 (2.3%)	8 (22%)	11	−36%, −4.0%	0.0009947*
EC CTh	3.40 ± 0.28	3.42 ± 0.27	3.36 ± 0.30	1.1	−0.05, 0.18	0.3
IT CTh	2.74 ± 0.11	2.74 ± 0.11	2.74 ± 0.11	0.36	−0.04, 0.05	0.7
EC cMD	1.30e-03 ± 1.40e-04 [8.70e-03;1.70e-03]	1.20e-03 ± 1.40e-04 [8.70e-04;1.60e-03]	1.30e-03 ± 1.40e-04 [1.00e-03;1.70e-03]	−1.5	0.00, 0.00	0.2
IT cMD	9.40e-04 ± 5.8e-05 [8.30e-04;1.20e-03]	9.30e-04 ± 6.3e-05 [8.30e-04;1.20e-03]	9.60e-04 ± 4e-05 [8.40e-04;1.10e-03]	−2.7	0.00, 0.00	0.008*
EC Tau	1.3 ± 0.3 [0.48;2.4]	1.2 ± 0.22 [0.48;1.8]	1.5 ± 0.36 [1;2.4]	−4.5	−0.42, −0.16	2.621733e-07*
IT Tau	1.4 ± 0.19 [0.74;2.2]	1.4 ± 0.16 [0.74;1.8]	1.5 ± 0.23 [1.2;2.2]	−3.5	−0.23, −0.06	0.001*
LMDR	16 ± 3.6 [7;24]	16 ± 3.6 [7;24]	16 ± 3.4 [8;21]	0.25	−1.2, 1.5	0.789
P.A.C.C.5	0.3 ± 0.75 [−1.9;2.4]	0.34 ± 0.75 [−1.9;2.4]	0.19 ± 0.75 [−1.5;1.3]	1.1	−0.14, 0.45	0.3

*P* value for this table were derived from comparisons between A− and A+ using *t* tests for continuous variables and chi-square tests for categorical values. A+/− Amyloid positive/negative, *cMD* cortical Mean Diffusivity, *CTh* Cortical Thickness, *IT* Inferior Temporal, *EC* entorhinal cortex, *APOE* apolipoprotein, *LMDR* Logical Memory Delayer Recall, *PACC5* Preclinical Alzheimer Cognitive Composite, *W* white, *NH* non-hispanic. *P* value = *t* test.

### MRI methods

All participants underwent a 3-Tesla MRI imaging procedure on a TimTrio scanner (Siemens, Erlangen, Germany) using a 12-channel phased-array head coil. Structural T1-weighted magnetization-prepared rapid-acquisition gradient-echo (MPRAGE; repetition time (TR) = 2200 ms, echo times (TE) = 1.54, or 3.36, or 5.18, or 7 ms, flip angle = 7°, 4× acceleration, 1.0 × 1.0 × 1.2 mm voxels), as well as DWI sequences, were registered. Structural segmentation was performed using FreeSurfer 6.0 (http://surfer.nmr.mgh.harvard.edu)^34^, and visually checked to correct potential processing errors. Cortical mean diffusivity was computed by processing DWI data using a surface-based approach combining FSL (FMRIB Software Library) (http://fsl.fmrib.ox.ac.uk/fsl/fslwiki, v5.0.9) and Freesurfer 6.0 (http://surfer.nmr.mgh.harvard.edu)^9^. Briefly, the methods sample the mean DTI metric in the mid-point of the cortical ribbon and sample it to each individual cortical surface reconstruction. The DTI processing details for this sample were explained in a previous publication^15^. Measures of cMD were computed based on baseline DWI data. For this study, similarly to our previous publication^15^, we focused on regional cMD assessed in two regions of interest (ROI): the entorhinal (EC) and inferior temporal (IT) cortices.

Structural MRI was processed for estimation of cortical thickness (CTh) using FreeSurfer 6.0^34^. Specifically, cortical segmentations were visually inspected to detect and correct processing errors and an automatic ROI parcellation was performed^35^. Similar to the cMD data, we focused on regional CTh assessed in the EC and inferior temporal IT cortices.

### PET methods

PET scans were performed on an ECAT EXACT HR+ scanner (Siemens, Erlangen, Germany) at the Massachusetts General Hospital PET facility. Acquisition parameters and methods have been previously detailed^15, 36,37^. PET images were co-registered to the participant’s native T1-MRI using FreeSurfer (mri_coreg).

FTP-PET values were corrected for partial volume (PVC) using the Geometric Transfer Matrix approach, and standardized uptake value ratios (SUVr) were obtained using the cerebellar gray matter as a reference. Based on previous literature, EC tau burden was selected as an early affected region in the course of AD^37,38^, and IT tau, a region in which tau accumulation has been proven to be characteristic of AD as opposed to normal aging^39,40^.

PiB-PET was computed in a cortical composite including frontal, lateral-temporal, parietal, and retrosplenial (PiB-FLR) regions^37^. Logan Distribution volume ratios (DVR) were computed using Logan models with the cerebellar gray matter as a reference. PVC was also applied. Participants were then assigned to two groups, amyloid-positive and negative, based on their baseline PiB-FLR DVR, using a 1.2 value (derived from a Gaussian mixture model) as a cutoff^41^. Out of 122 participants, our sample included 36 (29.5%) participants above this threshold who were considered amyloid-positive (A+).

### Memory measures

Episodic Memory (EM) was measured using the Logical Memory from the Wechsler Memory Scale^29^. The Logical Memory test is widely used in the context of AD in both clinical practice and research and consists of a paragraph read to the participant, who must recall as many relevant elements as possible, both immediately and after a delay (Delayed Recall, LMDR). The score ranges from 0 to 25, with a greater score meaning a better memory performance. The LMDR score was used for this study. Previous research found that CN participants with amyloidosis demonstrated a significantly greater LMDR decline than a control group^42^. As mentioned earlier, at screening, all participants had to perform in the normal range on the LMDR (adjusted for education).

### Statistics and reproducibility

We compared the two amyloid groups at baseline using t-tests and chi-square tests for continuous and categorical variables, respectively. Categorical variables included sex, *APOE*-ε4 carrier status, and race; continuous variables included age, education, LMDR, as well as brain regional FTP-PET and cMD values evaluated in both the EC and IT regions.

We performed two linear mixed-effects models to explore the effect of brain regional cMD at baseline on the longitudinal evolution of tau burden and EM performance. The first set of models used regional tau (FTP-PET) SUVr burden as the dependent variable, with one model for EC and another for IT tau. These models were corrected for age and sex and included the baseline amyloid group, baseline cMD value, and time (years from baseline), as well as the interaction between those three variables and all lower-order interaction terms, as fixed-effects independent variables, and incorporating random intercepts at the subject level. For each model, both the cMD and FTP-PET imaging measures were evaluated in the same ROI, i.e., the EC and IT, respectively. The models were computed as follows: Tau _EC or IT_ ~ Age + Sex + Baseline Tau _EC or IT_ + Baseline cMD _EC or IT_ × Baseline Amyloid Group × Time + (1|Subject).

The second set of models was similar, except that LMDR was included as the dependent variable. The formula was the following: LMDR ~ Age + Sex + Baseline LMDR + Baseline cMD _EC or IT_ × Baseline Amyloid Group × Time + (1|Subject). Type II likelihood ratio was used to test the main and interaction effects. P-values were corrected for multiple comparisons using the Bonferroni method^43^ for the four models. Effect sizes for the models were computed through Cohen’s f2 method^44^. Continuous values were standardized for the different predictors to be put on similar scales.

Finally, additional models were performed using baseline cortical thickness (CTh, using structural MRI measures), to investigate the specificity of the cMD results. That is, first we ran the following model: LMDR ~ Age + Sex + Baseline LMDR + Baseline CTh _EC or IT_ × Baseline Amyloid Group × Time + (1|Subject). Additionally, we ran the following model: LMDR ~ Age + Sex + CTh + Baseline LMDR + Baseline cMD _EC or IT_ × Baseline Amyloid Group × Time + (1|Subject). These results are presented as supplementary material.

Simple slope analyses (SSA)^45^ were conducted to assess the differential effects of baseline cMD in each group (A+ vs. A−). SSA is a post hoc analysis that can be used to help interpret significant interactions. When two or more terms interact, it means that the regression for each of them depends on the value of the other. SSA computes and examines the regressions using several values of the terms. For example, since our interactions consist of two continuous variables and one categorical (i.e., amyloid group), SSA was performed by computing the regression lines separately using the mean and standard deviation (SD) of cMD values (i.e., mean, −1SD, +1 SD) and the amyloid group over time. This procedure allows us to determine the directionality of each regression line in this three-way interaction.

All statistical analyses were performed using R4.1.0 (https://www.R-project.org/). Linear mixed-effect models were computed using the lme4 package^46^.

### Reporting summary

Further information on research design is available in the Nature Portfolio Reporting Summary linked to this article.

## Results

### Group comparisons at baseline

Our sample included 122 participants with 2.4 ± 0.55 (up to 4) follow-up observations. The majority were women (64.8%) with 16 ± 2.9 years of education; their baseline age was 71 ± 9.7 years old. Amyloid groups were equivalent on most demographic variables, including sex, education, race, and ethnicity (Table 1). However, A+ participants were significantly older than A− participants (76 ± 7.1 vs. 69 ± 9.9, *p* < 0.001). Among our sample, 37 (30.33%) participants were *APOE*-ε4 carriers, with a higher proportion of *APOE*-ε4 carriers seen in A+ participants when compared to A− participants (61.1% for A+ vs. 17.65% for A−, *p* < 0.001). At follow-up (mean number of follow-up visits = 2.4 ± 0.75), 8.2% (*N* = 10) of the participants had clinically progressed. There was a significant difference between the amyloid groups, with A+ participants demonstrating a higher proportion of progressed individuals then the A− group (*N* = 8/22% vs 2/2.3%, *p* < 0.001).

We did not find any statistically significant differences between amyloid groups for baseline LMDR, cortical thickness in the EC and IT regions, and baseline EC cMD (*p* values > 0.05). However, A+ participants showed greater IT cMD as well as tau burden (in both EC and IT regions) when compared to the A− group (all *p* < 0.004).

### Models predicting longitudinal FTP-PET

The first set of models aimed at predicting longitudinal tau increase over time, based on regional cMD values at baseline in A+ and A− participants in two models separately where: (1) both FTP-PET and baseline cMD were evaluated in the EC region, and (2) both FTP-PET and baseline cMD were assessed in the IT region (Table 2).

**Table 2:** Linear mixed-effects models predicting the longitudinal evolution of tauopathy and episodic memory.

	FTP-PET SUVr						Logical memory delayed recall					
	Entorhinal cortex			Inferior temporal cortex			Entorhinal cortex			Inferior temporal cortex		
	Est ± S.E. (CI95%)	E.S.	*p* values	Est ± S.E. (CI95%)	E.S.	*p* values	Est ± S.E. (CI95%)	E.S.	*p* values	Est ± S.E. (CI95%)	E.S.	*p* values
(Intercept)	−0.053 ± 0.038 (−0.128, 0.021)	0	0.640	−0.087 ± 0.041 (−0.169, −0.006)	0	0.142	0.154 ± 0.068 (0.019, 0.289)	0	0.103	0.155 ± 0.068 (0.02, 0.289)	0	0.098
Age (years)	−0.001 ± 0.03 (−0.061, 0.059)	−0.004	1.000	−0.084 ± 0.038 (−0.159, −0.009)	0.015	0.118	0.019 ± 0.048 (−0.077, 0.115)	0	1	0.072 ± 0.054 (−0.035, 0.178)	0.01	0.745
Sex (F)	0.011 ± 0.056 (−0.1, 0.123)	0	1	0.023 ± 0.061 (−0.098, 0.145)	0	1	−0.176 ± 0.102 (−0.378, 0.025)	0	0.343	−0.159 ± 0.1 (−0.356, 0.039)	0	0.456
Baseline	0.876 ± 0.029 (0.817, 0.934)	3.487	<2.2e-16*	0.888 ± 0.033 (0.823, 0.952)	2.774	<2.2e-16*	0.593 ± 0.046 (0.501, 0.685)	0.56	<2.2e-16*	0.597 ± 0.046 (0.506, 0.689)	0.57	<2.2e-16*
cMD	−0.003 ± 0.031 (−0.065, 0.059)	−0.004	1.000	−0.045 ± 0.036 (−0.116, 0.026)	0.004	0.836	−0.081 ± 0.054 (−0.188, 0.027)	0.005	0.557	−0.123 ± 0.056 (−0.235, −0.012)	0.016	0.123
Time (years)	0.051 ± 0.027 (−0.003, 0.104)	0.008	0.252	0.092 ± 0.031 (0.031, 0.153)	0.025	0.013*	0.217 ± 0.029 (0.161, 0.273)	0.059	2.058e-07*	0.21 ± 0.029 (0.154, 0.267)	0.053	3.793e-07*
Baseline amyloid group (PiB)	0.151 ± 0.062 (0.028, 0.274)	0.023	0.066	0.24 ± 0.067 (0.107, 0.373)	0.047	0.002*	−0.27 ± 0.105 (−0.477, −0.063)	0.024	0.044*	−0.254 ± 0.106 (−0.463, −0.044)	0.021	0.073
cMD × time	−0.031 ± 0.026 (−0.083, 0.021)	0	0.985	−0.059 ± 0.026 (−0.111, −0.007)	0	0.107	−0.063 ± 0.029 (−0.12, −0.007)	0	0.115	−0.061 ± 0.027 (−0.114, −0.009)	0	0.091
cMD × baseline amyloid group	0.129 ± 0.058 (0.014, 0.244)	0.018	0.112	0.085 ± 0.081 (−0.077, 0.246)	0.001	1	−0.124 ± 0.098 (−0.318, 0.071)	0.004	0.840	−0.175 ± 0.127 (−0.426, 0.076)	0.004	0.680
Time × baseline amyloid group	0.149 ± 0.048 (0.054, 0.243)	0.029	0.009*	0.269 ± 0.057 (0.156, 0.381)	0.069	3.656e-07*	−0.23 ± 0.047 (−0.323, −0.138)	0.023	7.951e-08*	−0.197 ± 0.05 (−0.295, −0.099)	0.015	7.328e-07*
cMD × time × baseline amyloid group	0.135 ± 0.052 (0.031, 0.238)	0.019	0.044*	0.052 ± 0.069 (−0.084, 0.188)	−0.001	1	−0.149 ± 0.05 (−0.249, −0.05)	0.006	0.013*	−0.163 ± 0.066 (−0.292, −0.033)	0.003	0.055

*cMD* cortical Mean Diffusivity, *PiB* PET Amyloid Tracer Positivity status.

**Fig. 1:**
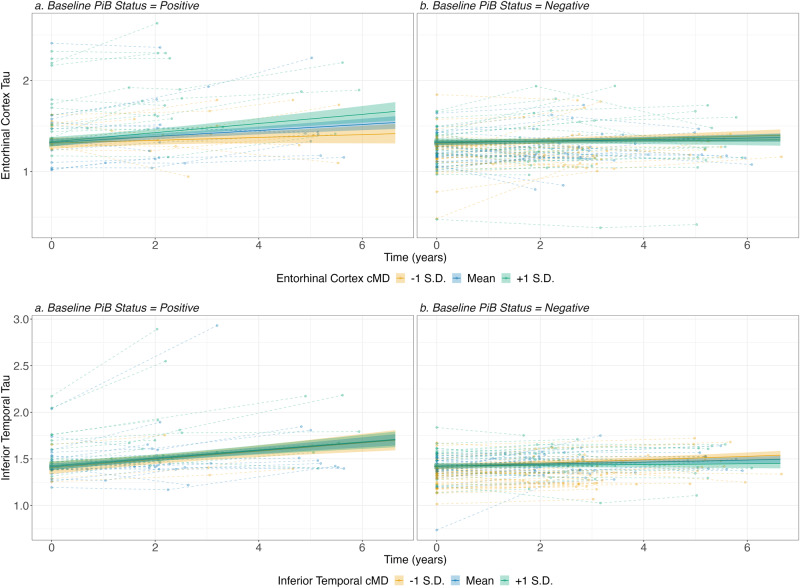
Impact of entorhinal and inferior temporal cMD on longitudinal regional tau across amyloid groups. The figure displays results from the models using either EC (I.a and I.b) or IT (II.a and II.b) cMD in interaction with time and amyloid burden. Amyloid was dichotomized, i.e., split into amyloid-positive (I.a. and II.a.) vs negative (I.b. and II.b.) based on a computed threshold using the cerebellar gray matter as reference. cMD cortical mean diffusivity, PiB 11C-Pittsburgh Compound-B, S.D. standard deviation.

We observed a significant three-way interaction of amyloid status, baseline EC-cMD, and time in the model predicting EC-Tau (*β* = 0.135 ± 0.052, CI = 0.031–0.238, ES = 0.019, *p* = 0.044; see Fig. 1, top-row, as well as Table 2). The model did not show other significant interactions nor a main effect of time. A significant interaction was also observed in both models between time and amyloid status (EC *β* = 0.149 ± 0.048, CI = 0.054–0.243, ES = 0.029, *p* = 0.009; IT *β* = 0.269 ± 0.057, CI = 0.156–0.381, ES = 0.069, *p* < 0.001).

To further understand the relationship between baseline EC-cMD and EC-tau over time in the amyloid-positive and negative groups, SSA were performed (Table 3). The results showed a significant increase for A− with low baseline cMD values (*β* = 0.08 ± 0.04, CI = 0.01–0.16, ES = 0.09 *p* = 0.032) but no significant effect for higher values. In A+ participants, low baseline cMD values were not associated with greater tau accumulation over time. However, A+ participants demonstrating greater levels of baseline cMD, showed a significant increase of tau over time (*β* = 0.2 ± 0.004, CI = 0.12–0.28, ES = 0.09; and *β* = 0.3 ± 0.006, CI = 0.19–0.42, ES = 0.09; both *p* < 0.001).

**Table 3:** Simple slopes analyses for the three-way interactions time × baseline PiB status × cMD.

Model	cMD	Entorhinal cortex		Inferior-temporal cortex	
		A−	A+	A−	A+
Tau	−1sd	0.08 ± 0.04 ([0.01;0.16], ES = 0.09, *p* = 0.032*)	0.1 ± 0.06 ([−0.03;0.22], ES = 0.09, *p* = 0.132)	0.15 ± 0.04 ([0.07;0.23], ES = 0.09, *p* = 0.00013*)	0.37 ± 0.09 ([0.19;0.55], ES = 0.09, *p* = 7.537289e-05*)
	Mean	0.05 ± 0.03 ([0;0.1], ES = 0.09, *p* = 0.063)	0.2 ± 0.04 ([0.12;0.28], ES = 0.09, *p* = 1.377553e-06*)	0.09 ± 0.03 ([0.03;0.15], ES = 0.09, *p* = 0.003*)	0.36 ± 0.05 ([0.26;0.46], ES = 0.09, *p* = 2.823435e-12*)
	+1sd	0.02 ± 0.04 ([−0.05;0.09], ES = 0.09, *p* = 0.602)	0.3 ± 0.06 ([0.19;0.42], ES = 0.09, *p* = 3.660979e-07*)	0.03 ± 0.04 ([−0.05;0.12], ES = 0.09, *p* = 0.431)	0.35 ± 0.07 ([0.22;0.48], ES = 0.09, *p* = 2.117439e-07*)
	−1sd	0.28 ± 0.04 ([0.2;0.36], ES = 0.02, *p* = 1.023953e-11*)	0.2 ± 0.06 ([0.08;0.32], ES = 0.02, *p* = 1.099001e-03*)	0.27 ± 0.04 ([0.2;0.35], ES = 0.01, *p* = 2.345607e-12*)	0.24 ± 0.09 ([0.07;0.41], ES = 0.01, *p* = 0.006*)
LMDR	Mean	0.22 ± 0.03 ([0.16;0.27], ES = 0.02, *p* = 4.790351e-14*)	−0.01 ± 0.04 ([−0.09;0.06], ES = 0.02, *p* = 0.725)	0.21 ± 0.03 ([0.15;0.27], ES = 0.01, *p* = 4.029126e-13*)	0.01 ± 0.04 ([−0.07;0.09], ES = 0.01, *p* = 0.739)
	+1sd	0.15 ± 0.04 ([0.07;0.23], ES = 0.02, *p* = 1.315426e-04*)	−0.23 ± 0.05 ([−0.32;−0.13], ES = 0.02, *p* = 7.397033e-06*)	0.15 ± 0.04 ([0.07;0.23], ES = 0.01, *p* = 2.141936e-04*)	−0.21 ± 0.06 ([−0.32;−0.1], ES = 0.01, *p* = 0.0002*)

Estimate ± standard-error (confidence interval low; high, effect size, *p* value); cMD cortical Mean Diffusivity, *A+/−* Amyloid positive/negative using PiB as a PET Amyloid Tracer Positivity status, *LMDR* Logical Memory Delayed Recall.

The model investigating whether baseline IT-cMD, and time predicted IT-Tau was not significant (see Fig. 1, bottom-row, and Table 2).

### Models predicting longitudinal memory

Two models predicting the longitudinal evolution of EM using the LMDR value as the dependent variable were performed, using EC and IT cMD values at baseline as independent predictors, respectively (Table 2). The models showed a significant and positive main effect of baseline LMDR value in the models (EC *β* = 0.593 ± 0.046, CI = 0.501–0.685, ES = 0.56, *p* < 0.001; IT *β* = 0.597 ± 0.046, CI = 0.506–0.689, ES = 0.57, *p* < 0.001). In both models, the main effect of time was significant (*p* < 0.001) but didn’t show a main effect of baseline cMD.

**Fig. 2:**
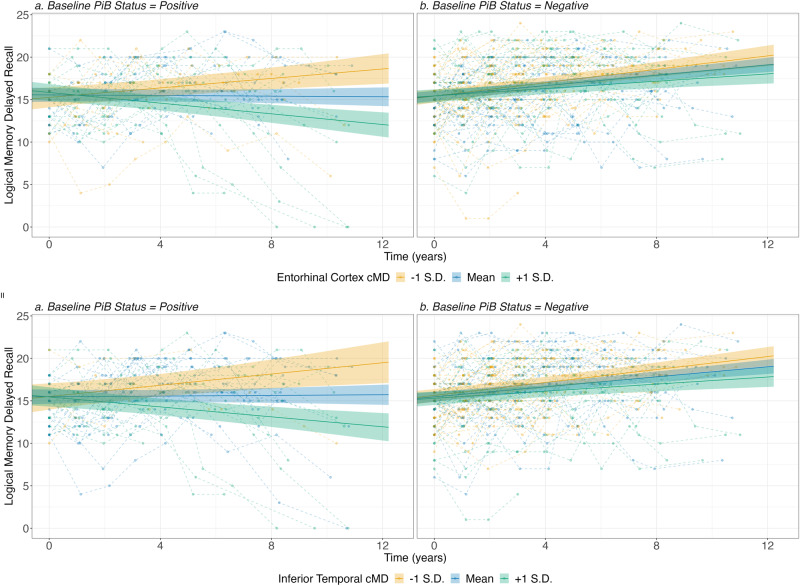
Impact of entorhinal and inferior temporal CTh on longitudinal regional tau across amyloid groups. Similar to figure one, predicting the EM evolution based on the interaction between amyloid and MTL-cMD over time. cMD cortical mean diffusivity, PiB 11C-Pittsburgh Compound-B, S.D. standard deviation.

We observed a three-way interaction between amyloid status, baseline cMD value, and time in the EC model (*β* = −0.149 ± 0.05, CI = −0.249–−0.05, ES = 0.006, *p* = 0.013), and a trending effect in the IT model (*β* = −0.163 ± 0.066, CI = −0.292–−0.033, ES = 0.003, *p* = 0.055, see Table 2 and Fig. 2). When computing the simple slopes post hoc analyses, both models showed the same pattern (Table 3). That is, in the A− group, we observed improving memory performances over time, with greater baseline cMD associated with a smaller improvement over time. In the A+ group, we also observe an increase in lower baseline cMD values (*β* = 0.2 ± 0.06, CI = 0.08–0.32, ES = 0.02, and *β* = 0.24 ± 0.09, CI = 0.07–0.41, ES = 0.01, for EC and IT, respectively, both *p* < 0.01). However, in A+ participants, greater baseline cMD was associated with a decline in LMDR over time (*β* = −0.23 ± 0.05, CI = −0.32–−0.13, ES = 0.02 for EC and *β* = −0.21 ± 0.06, CI = −0.32–−0.01, ES = 0.01 for IT, both *p* < 0.001, see Fig. 2I.b. and II.b.). Both models also showed significant effects of the interactions between amyloid status and time, as well as between baseline cMD and time (all *p* < 0.001).

## Discussion

Previous studies have shown that brain microstructural changes, measured with cMD, could be observed in early preclinical AD stages and are related to brain tauopathy burden as measured using tau PET imaging cross-sectionally^9, 10,15^. However, the association between baseline cMD and the longitudinal accumulation of tau burden in the brain is unknown. Our current study investigated the longitudinal association between early cMD at baseline and longitudinal evolution of regional tau accumulation and episodic memory performance in cognitively asymptomatic participants at risk for AD as defined by their amyloid status. We found a significant impact of regional cortical diffusivity on temporal tau across time, with an increased effect observed in the A+ participants compared to the A− participants. Similarly, the episodic memory models showed that increased cortical mean diffusivity in the medial-temporal lobe was associated with steeper EM decline, especially in the A+ group. These results support the consideration of cMD measures as potential early sensitive and non-invasive biomarkers of disease progression in individuals at risk for AD.

Our study focused on cMD and tau PET imaging measures in two specific brain ROIs, i.e., EC and IT. Although being present almost universally with aging^47^, tau accumulation can be found at especially high concentrations in AD^1–3,48^, often following a well-known topographical pattern of accumulation^49^. Some regions are known to be affected explicitly in AD compared to normal aging, including the IT region^39,40^. Likewise, following Braak’s stages, it is documented that the EC region is among the first to be affected^37,38^. In our sample, we included older participants, all CN at baseline, and compared them using their baseline amyloid status. Over the course of AD, amyloidosis would precede tauopathy^1,50^. However, studies noted a synergistic effect of these two biomarkers^51^, with amyloid accelerating the tau accumulation. The observation that our amyloid-positive individuals had significantly increased tau pathology as compared to the amyloid-negative individuals at baseline is in line with this notion. Additionally, at baseline, our sample did not show significant differences in cognition between amyloid groups. During the preclinical stage, subtle cognitive changes occur, especially in EM and executive function, and have been associated with AD biomarkers^24,52,53^. These changes are usually referred to as “subtle cognitive decline”^3^, i.e., a progressive decline that is not sufficient to be detected at a single visit level, explaining the absence of differences in our baseline comparison.

When modeling tau accumulation with time as predicted by the participant’s baseline regional cMD in both amyloid groups, we found a significant three-way interaction between amyloid status, time, and cMD in the EC region (*p* = 0.044), but a non-significant level when looking at the IT region (see Fig. 1). Both of these models showed that, in amyloid-negative participants, greater levels of regional cMD did not have an impact on longitudinal tau levels locally. However, for the EC-cMD model, amyloid-positive participants demonstrated different magnitude of tau accumulation depending on their baseline EC-cMD, with higher cMD associated with greater tau accumulation over time. In contrast, individuals with elevated amyloid and low-cMD at baseline did not demonstrate significant tau accumulation over time. A previous cross-sectional study showed that increased cMD in a set of specific brain regions was associated with greater tau levels but not with amyloid burden^15^. Similarly, other studies confirmed that the presence of AD biomarkers (i.e., both amyloid and tau) was associated with increased regional cMD^9^. One study, using a pseudo-longitudinal model of cMD in AD based on estimated years before the onset of clinical symptoms, showed that preclinical AD participants have a decrease followed by an increase in cMD in later stages^10^. Our current study confirms the previously reported positive association between cMD and tau burden cross-sectionally^15^ and further extends the findings to show that this relationship can also be observed concerning the longitudinal accumulation of tau in cognitively asymptomatic older adults with elevated amyloid but not in those without elevated amyloid. Our findings advocate for the specificity of cMD increased diffusivity in AD-sensitive areas for CN participants at risk for AD. This result also corroborates the previous proposal that a synergistic interaction between amyloid and tau determines the evolution of AD, and that the first trigger**s** the toxicity of the second^51,54,55^.

In previous work, tau^56,57^ and amyloid burden^24,52,53^ have been linked to cognitive decline in the early stages of AD. To explore the longitudinal relationship between regional cMD and episodic memory decline in early AD, we implemented statistical models predicting EM decline as a function of baseline cMD in both amyloid groups.

EC-cMD showed a significant three-way interaction, in the form of a “fan-effect”, with amyloid in predicting cognition over time, and a trend was found in the IT-cMD model. Overall, participants with low baseline cMD showed small but significant episodic memory improvement over time. In the A− participants, high-baseline cMD was associated with an increase over time. However, in the A+ group, high-baseline cMD was related to a significantly steeper memory decline over time (see Fig. 2). The longitudinal increase observed in A− participants, as well as the A+-low-cMD participants, can be interpreted as a ‘practice effect,’ i.e., the advantage of having been previously exposed to similar testing material for subsequent performance. Previous studies have shown that, while the practice effect exists in the preclinical AD context, it is reduced compared to controls^58^. The current findings of decreased memory performance only in the A+ high cMD participants further support the notion that altered cMD is an early marker of EM decline in preclinical participants.

This three-way interaction also confirms the similar relationship between medial-temporal cMD and a measure of global cognition (the Preclinical Alzheimer’s disease Cognitive Composite, PACC) that was previously found^15^. The scores on which the PACC is built are primarily, but not exclusively, associated with EM^59^, a cognitive function consistently related to MTL functioning^60^, and integrity^61^. The PACC measure, as a composite score, have the advantage of being very efficient in describing longitudinal evolution and predicting progression in cohort studies. However, the PACC is not able to measure EM evolution itself, as it is using measures also related to executive functioning. In addition, it cannot describe an individual’s evolution outside of a cohort study (e.g., in a clinical setting), as the measure itself uses the mean and standard deviation of the entire group to create the measure. The current study focused on EM, using the LMDR, an EM test widely used in both clinical practice and research. The models predicting tau and our findings showing that baseline regional EC-cMD predicted tau increase over time in CN participants with elevated amyloid serve as a potential explanation for the indirect effect of MTL dysfunction caused by tau accumulation and subsequent atrophy. Accordingly, previous research has linked AD patients’ cognitive performance, and especially EM, with tau accumulation in the MTL^57,62^. Our results suggest that amyloid-positive participants exhibit greater tau accumulation in the MTL over time, leading to regional cortical thinning, dysfunction, and ultimately a greater EM decline.

The contribution of amyloid to longitudinal cognitive decline has been the topic of much research, and our results support a critical body of work. In our study, greater memory decline was observed in amyloid-positive participants as compared to amyloid-negative participants, and a significant interaction between amyloid status and time in both memory models was observed. Previous studies observed that the pattern of both amyloid and tau accumulation overlapped with the default mode network^16^. Consequently, it has been proposed that brain amyloid accumulation could affect the functions supported by this network, including executive function and episodic memory^18,19^.

Our results also showed an absence of sex effect on cognition. Previous research has shown sex differences in the episodic memory profile of MCI and AD dementia patients^63^. More recently, it has been shown that CN female participants with high amyloid load demonstrate greater cognitive decline than male participants^64^. In our current study, the absence of a sex effect may have several explanations. Future models are needed to answer this question more in detail.

We also ran supplementary analyses looking at cortical thickness, rather than cMD and found a significant interaction in the EC, but not in the IT, such that decreased CTh at baseline predicted a faster decline in EM (Supp. Tab. 1). More importantly, when adding CTh as a covariate in the cMD models, the three-way interaction between cMD, time and baseline amyloid status remained significant for both EC and IT cMD (Supp. Tab. 2). These results are in line with our previous findings investigating cognition using a cognitive composite score (PACC) as our main outcome variable^15^, and provide further evidence that cMD independently explains variance in cognitive decline, beyond cortical thickness in these regions.

### Limitations

This study has several limitations. First, our current sample, highly educated and predominantly white, is not representative of the global population. Also, the relatively short follow-up period could prevent us from observing some changes, both in tau and cognition, in the context of preclinical AD. Similarly, this also prevented us to include random slopes in the models. Subsequently, longer follow-up time and with more data, is needed to confirm our observations in a more diverse population with random slopes included.

In this research, we used two MTL regions for tau (EC and IT). MTL functioning and integrity, especially in AD, have been mostly related to EM performance^60^. However, episodic memory is not the only measure that has shown a subtle decline over the course of the preclinical AD phase^3^. Other cognitive domains may involve a distinct network^61^ that does not necessarily include the MTL. Further research involving other brain regions and/or cognitive functions might show some interesting results.

In this current study, we used baseline cMD to predict future tau accumulation. However, future studies should also explore the longitudinal evolution of tau and cMD, including longitudinal paired DWI measures along with tau scans over time.

## Conclusion

Our results show a relationship between baseline cMD and longitudinal accumulation of tau in CN participants at risk for AD. This was particularly the case in the entorhinal cortex, a region known to be affected very early over the course of AD^37,38^. The same relationship was observed with episodic memory decline, showing that CN participants at risk for AD have greater memory decline over time associated with higher baseline diffusivity. These results confirm previous cross-sectional^12^ as well as findings obtained with cMD and longitudinal global cognition (PACC)^15^.

As compared to tau PET tracers, DTI-MRI does not entail radiation exposure and is used more widely in the clinical setting. Therefore, these results support the use of this technique, along with other methods such as PET or *APOE* genotyping, as an efficient early screening biomarker.

## Supplementary information

Supplemental Material

Reporting Summary

## Data Availability

Data are publicly available upon request and agreement to our data use agreement (DUA). The DUA and the data request form can be accessed at: https://habs.mgh.harvard.edu/.
