# Supplementary material for: Cortical microstructural changes predict tau accumulation and episodic memory decline in older adults harboring amyloid
Source: Commun Med (Lond). 2023 Aug 1;3:106. doi: 10.1038/s43856-023-00324-7 (PMC10394044; doi:10.1038/s43856-023-00324-7)

Supplementary Material

Supplementary Table 1. Linear mixed-effects models using cortical thickness.

	Logical Memory Delayed Recall					
	Entorhinal Cortex			Inferior Temporal Cortex		
	**Est ± S.E. (CI95%)**	**E.S.**	**p-values**	**Est ± S.E. (CI95%)**	**E.S.**	**p-values**
(Intercept)	0.151 ± 0.069 (0.015, 0.287)	0	0.121	0.151 ± 0.071 (0.011, 0.292)	0	0.139
Age (years)	0.019 ± 0.05 (-0.079, 0.118)	0.001	1	0.016 ± 0.052 (-0.088, 0.12)	0	1
Sex (F)	-0.159 ± 0.101 (-0.359, 0.042)	0	0.478	-0.147 ± 0.103 (-0.352, 0.057)	0	0.625
Baseline	0.587 ± 0.046 (0.495, 0.679)	0.57	<0.001*	0.588 ± 0.049 (0.49, 0.686)	0.507	<0.001*
CTh	0.053 ± 0.061 (-0.068, 0.174)	0.003	1	0.001 ± 0.061 (-0.12, 0.121)	-0.001	1
Time (years)	0.219 ± 0.029 (0.162, 0.277)	0.055	<0.001*	0.226 ± 0.03 (0.167, 0.285)	0.057	<0.001*
Baseline Amyloid Group (PiB)	-0.292 ± 0.105 (-0.501, -0.084)	0.028	0.025*	-0.313 ± 0.107 (-0.526, -0.1)	0.031	0.018*
CTh x Time	0.073 ± 0.028 (0.017, 0.129)	0	0.043*	-0.002 ± 0.032 (-0.065, 0.06)		1.000
CTh x Baseline Amyloid Group	0.103 ± 0.1 (-0.096, 0.301)	0.002	1	0.043 ± 0.099 (-0.153, 0.239)	0	1
Time x Baseline Amyloid Group	-0.243 ± 0.047 (-0.335, -0.151)	0.025	<0.001*	-0.274 ± 0.048 (-0.369, -0.18)	0.031	<0.001*
CTh x Time x Baseline Amyloid Group	0.171 ± 0.046 (0.081, 0.262)	0.012	0.001*	0.112 ± 0.048 (0.018, 0.206)	0.005	0.078

Supplementary Table 2. Linear mixed-effects models predicting Logical Memory Delayed Recall corrected for cortical thickness.

	Logical Memory Delayed Recall					
	Entorhinal Cortex			Inferior Temporal Cortex		
	**Est ± S.E. (CI95%)**	**E.S.**	**p-values**	**Est ± S.E. (CI95%)**	**E.S.**	**p-values**
Age (years)	0.04 (-0.05;0.12)	0.005	0.386	0.06 (-0.03;0.16)	0.010	0.175
Sex (F)	-0.18 (-0.36;0.01)	0	0.34*	-0.17 (-0.34;-0.01)	0	0.40*
Baseline	0.64 (0.56;0.71)	0.559	<0.001*	0.63 (0.55;0.71)	0.533	<0.001*
CTh	-0.03 (-0.12;0.05)	-0.005	0.399	-0.01 (-0.09;0.07)	-0.001	0.815
cMD	-0.07 (-0.20;0.05)	0	0.478	-0.10 (-0.22;0.02)	0.012	0.129
Time (years)	0.20 (0.11;0.29)	0.058	0.004*	0.20 (0.11;0.30)	0.056	0.003
Baseline Amyloid Group (PiB)	-0.28 (-0.51;-0.05)	0.027	0.454	-0.25 (-0.48;-0.02)	0.022	0.345
cMD x Time	-0.04 (-0.13;0.06)	0	0.008*	-0.04 (-0.013;0.04)	0	0.025*
cMD x Baseline Amyloid Group	-0.15 (-0.37;0.07)	0.013	0.607	-0.24 (-0.51;0.004)	0.010	0.798
Time x Baseline Amyloid Group	-0.22 (-0.38;-0.07)	0.029	0.001*	-0.19 (-0.36;-0.03)	0.022	0.002*
cMD x Time x Baseline Amyloid Group	-0.21 (-0.37;-0.04)	0.026	0.013*	-0.25 (-0.45;-0.04)	0.012	0.017*

Supplementary Figure 1. Longitudinal impact of CTh on longitudinal Episodic Memory

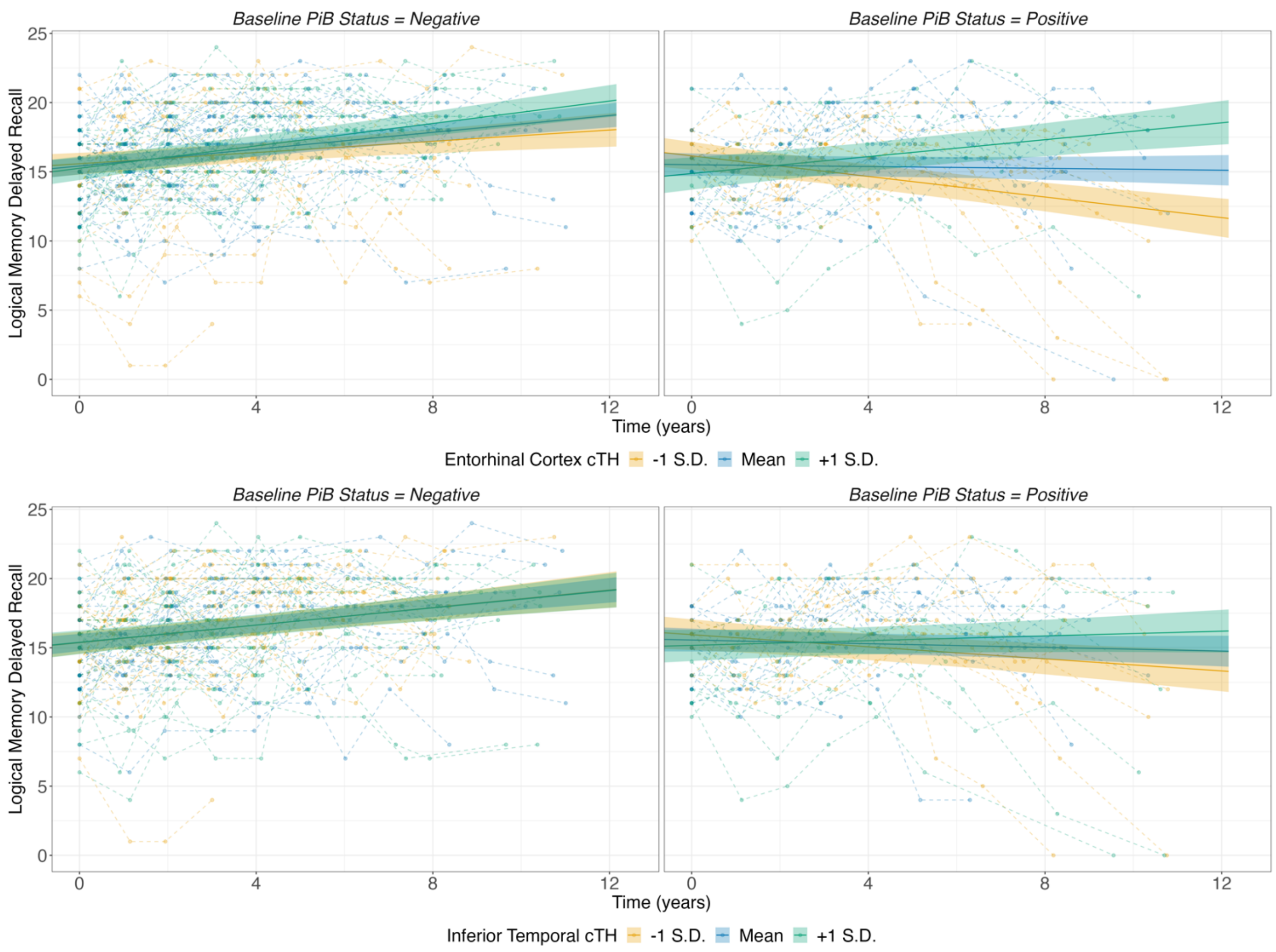

Supplementary Figure 2. Longitudinal impact of cMD on longitudinal Episodic Memory, corrected for CTh.

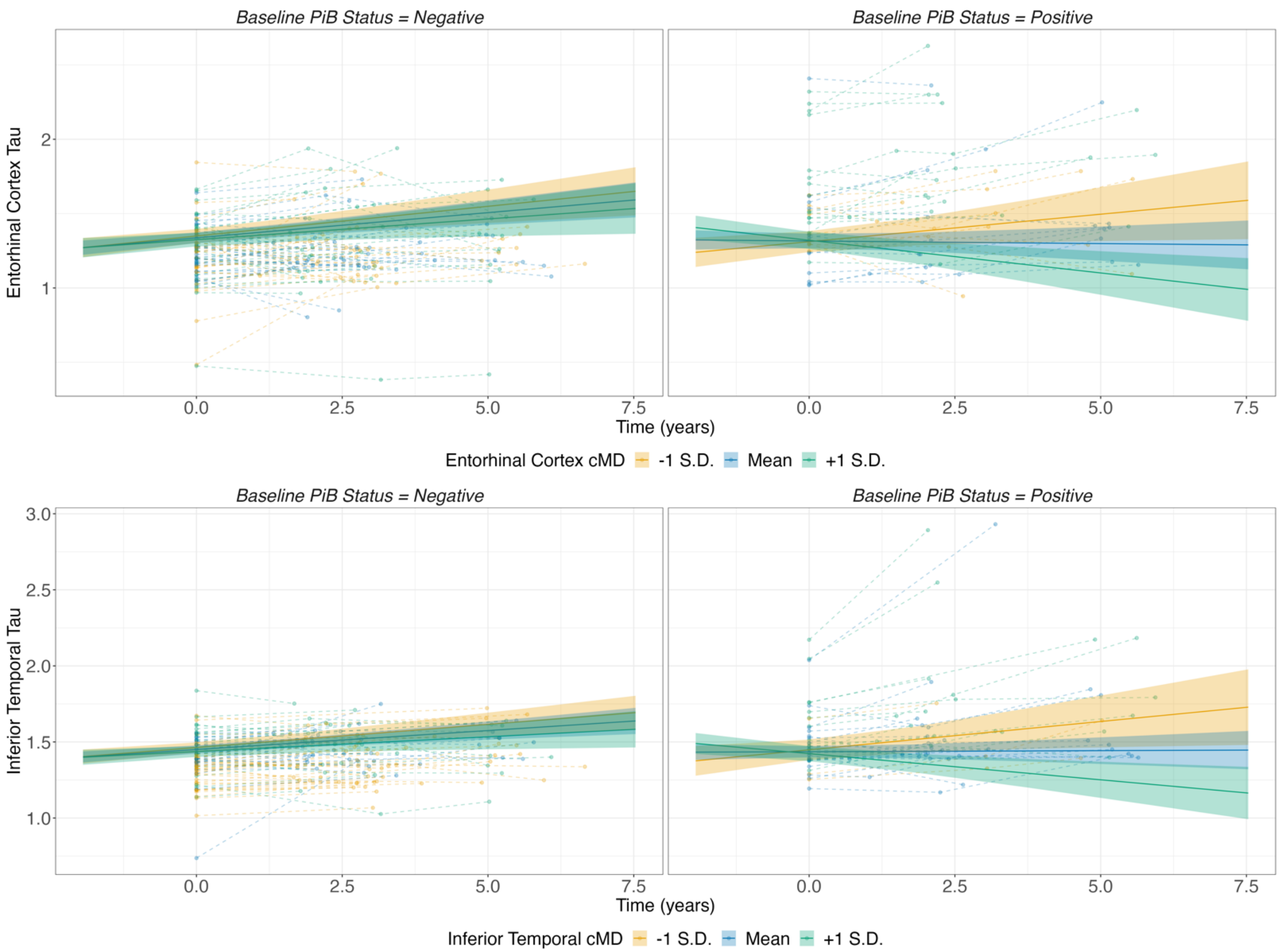

Supplement: Supplementary file 1 — Supplemental Material [file 43856_2023_324_MOESM1_ESM.pdf]
