# Supplementary material for: Cortical microstructural changes predict tau accumulation and episodic memory decline in older adults harboring amyloid
Source: Commun Med (Lond). 2023 Aug 1;3:106. doi: 10.1038/s43856-023-00324-7 (PMC10394044; doi:10.1038/s43856-023-00324-7)
Supplement: Supplementary file 2 — Reporting Summary [file 43856_2023_324_MOESM2_ESM.pdf]

nature portfolio

Corresponding author(s): Patrizia Vannini
Last updated by author(s): 2023/05/30

Reporting Summary

Nature Portfolio wishes to improve the reproducibility of the work that we publish. This form provides structure for consistency and transparency in reporting. For further information on Nature Portfolio policies, see our Editorial Policies and the Editorial Policy Checklist.

Statistics

For all statistical analyses, confirm that the following items are present in the figure legend, table legend, main text, or Methods section.

n/a	Confirmed	
☐	☒	The exact sample size (*n*) for each experimental group/condition, given as a discrete number and unit of measurement
☐	☒	A statement on whether measurements were taken from distinct samples or whether the same sample was measured repeatedly
☐	☒	The statistical test(s) used AND whether they are one- or two-sided<br>*Only common tests should be described solely by name; describe more complex techniques in the Methods section.*
☐	☒	A description of all covariates tested
☐	☒	A description of any assumptions or corrections, such as tests of normality and adjustment for multiple comparisons
☐	☒	A full description of the statistical parameters including central tendency (e.g. means) or other basic estimates (e.g. regression coefficient) AND variation (e.g. standard deviation) or associated estimates of uncertainty (e.g. confidence intervals)
☐	☒	For null hypothesis testing, the test statistic (e.g. *F*, *t*, *r*) with confidence intervals, effect sizes, degrees of freedom and *P* value noted<br>*Give P values as exact values whenever suitable.*
☒	☐	For Bayesian analysis, information on the choice of priors and Markov chain Monte Carlo settings
☒	☐	For hierarchical and complex designs, identification of the appropriate level for tests and full reporting of outcomes
☐	☒	Estimates of effect sizes (e.g. Cohen's *d*, Pearson's *r*), indicating how they were calculated

Our web collection on statistics for biologists contains articles on many of the points above.

Software and code

Policy information about availability of computer code

Data collection	Data have been collected in-person, or using the REDCap, a secure web application for online surveys and databases.<br>Structural segmentation was performed using FreeSurfer 6.0 (http:// surfer.nmr.mgh.harvard.edu), and visually checked to correct potential processing errors. Cortical mean diffusivity was computed by processing DWI data using a surface-based approach combining FSL (FMRIB Software Library) (http://fsl.fmrib.ox.ac.uk/fsl/fslwiki, v5.0.9) and Freesurfer 6.0 9.
Data analysis	All the data analyses have been performed using R, an open source statistical software, in it's 4.1.0 version.

For manuscripts utilizing custom algorithms or software that are central to the research but not yet described in published literature, software must be made available to editors and reviewers. We strongly encourage code deposition in a community repository (e.g. GitHub). See the Nature Portfolio guidelines for submitting code & software for further information.

Data

Policy information about availability of data

All manuscripts must include a data availability statement. This statement should provide the following information, where applicable:
- Accession codes, unique identifiers, or web links for publicly available datasets
- A description of any restrictions on data availability
- For clinical datasets or third party data, please ensure that the statement adheres to our policy

Data from the Harvard Aging Brain Study (HABS) are available upon request at: https://habs.mgh.harvard.edu/

Human research participants

Policy information about studies involving human research participants and Sex and Gender in Research.

Reporting on sex and gender	The Sex variable has been collected and used in our analyses as a covariate along with other variables considered as "demographic variables".
Population characteristics	Participants were enrolled in the HABS cohort if they were deemed cognitively normal at baseline, based on a Clinical Dementia Rating (CDR) evaluation and performance on the Logical Memory delayed recall score, Mini-Mental State Examination (MMSE) and Geriatric Depression Scale (GDS). Our current sub-sample (n=122) has been selected based on that all participants had underdone brain imaging, including structural MRI (T1-weighted and DWI), as well as PET with amyloid (PIB) and tau (flortaucipir [FTP]) tracers. All assessments were performed within one year of the MRI scan. In addition, all participants had at least one longitudinal assessment, including MRI, FTP-PET, and cognition (in addition to the baseline visit).
Recruitment	Participants were selected from the Harvard Aging Brain Study (HABS; https://habs.mgh.harvard.edu), a single-center observational study conducted at the Massachusetts General Hospital. HABS is a longitudinal study focused on older adults, cognitively normal at enrollment, aiming to improve our understanding of AD's preclinical stages. Over the course of the HABS study this far, we have noticed that our participants tend to have slightly higher than average educational attainment and there are slightly more women than men. These characteristics of the sample may impact the generalizablity of the results from HABS.
Ethics oversight	The protocol was approved by the Mass General Brigham institutional review board, and study procedures were carried out only after participants reviewed and signed the consent form

Note that full information on the approval of the study protocol must also be provided in the manuscript.

Field-specific reporting

Please select the one below that is the best fit for your research. If you are not sure, read the appropriate sections before making your selection.

☒ Life sciences ☐ Behavioural & social sciences ☐ Ecological, evolutionary & environmental sciences

For a reference copy of the document with all sections, see nature.com/documents/nr-reporting-summary-flat.pdf

Life sciences study design

All studies must disclose on these points even when the disclosure is negative.

Sample size	This is a longitudinal cohort study and considering the novelty of the cortical mean diffusivity and longitudinal tau PET data, a power analysis was not possible. However, taking into account previous work from our group, we were confident that n=122 at baseline, all of whom had at least two longitudinal tau PET timepoints, would be sufficient to test for an association between cortical mean diffusivity at baseline and the accumulation of tau burden and cognitive decline.
Data exclusions	In the Harvard Aging Brain Study (HABS), participants are excluded at study entry if they had a history of alcoholism, drug abuse, or head trauma.
Replication	No formal replication was done.
Randomization	NA. No randomization was used. This is a cohort study.
Blinding	NA. However, the Clinical Dementia Rating (CDR) was completed by neuropsychologists and psychiatrists and rated independently from other cognitive test results, and all CDR raters are blinded to participant biomarker status.

Reporting for specific materials, systems and methods

We require information from authors about some types of materials, experimental systems and methods used in many studies. Here, indicate whether each material, system or method listed is relevant to your study. If you are not sure if a list item applies to your research, read the appropriate section before selecting a response.

Materials & experimental systems

n/a	Involved in the study	
☒	☐	Antibodies
☒	☐	Eukaryotic cell lines
☒	☐	Palaeontology and archaeology
☒	☐	Animals and other organisms
☒	☐	Clinical data
☒	☐	Dual use research of concern

Methods

n/a	Involved in the study	
☒	☐	ChIP-seq
☒	☐	Flow cytometry
☐	☒	MRI-based neuroimaging

Magnetic resonance imaging

Experimental design

Design type: Only structural MRI and DWI. No functional MRI was performed.

Design specifications: Structural MRI sequences were acquired on a Siemens 3T Trio Tim MRI. Full details of our MRI protocol can be found at our data-sharing site: https://habs.mgh.harvard.edu/wp-content/uploads/2020/06/ADNI2_T1.pdf

Behavioral performance measures: NA

Acquisition

Imaging type(s): Structural and diffusion-weighted MRI

Field strength: 3 Tesla

Sequence & imaging parameters: The T1-weighted MPRAGE structural images were collected using the following scan parameters: repetition time (TR) = 2200 ms, echo times (TE) = 1.54, or 3.36, or 5.18, or 7 ms, flip angle = 7°, 4x acceleration, 1.0 x 1.0 x 1.2 mm voxels

Area of acquisition: Whole brain scan

Diffusion MRI: ☒ Used ☐ Not used

Parameters: A single shot spin echo planar imaging sequence was used with the following parameters: TR=6230, TE=84ms, a flip angle of 90°, FOV: 256 x 256 x 128 mm3; acquired isotropic voxel size 2 mm3, 30 isotropically distributed diffusion-sensitizing gradients with a b-value of 700 s/mm2 and five non-diffusion weighted images (b = 0 s/mm2)

Preprocessing

Preprocessing software: Structural MRI preprocessing steps were performed using FreeSurfer 6.0 (http://surfer.nmr.mgh.harvard.edu), and visually checked to correct potential processing errors. DWI data were preprocessed using FSL (FMRIB Software Library) (http://fsl.fmrib.ox.ac.uk/fsl/fslwiki, v5.0.9) and Freesurfer 6.0 9.

Normalization: DTI data was corrected for echo-planar imaging susceptibility by applying an affine registration to the skull-stripped T1 image of FreeSurfer.

Normalization template: Cortical mean diffusivity maps were normalized to a standard surface template from Freesurfer (fsaverage) and smoothed using a 15-mm 2D full-width half-maximum Gaussian kernel across the cortical mantle.

Noise and artifact removal: The standard preprocessing pipeline of FreeSurfer incorporates motion correction, intensity correction and skull-stripping. Diffusion data were corrected for eddy-current distortions, subject motion and echo-planar imaging susceptibility using FSL software.

Volume censoring: NA

Statistical modeling & inference

Model type and settings: We performed univariate statistical analyses by applying linear mixed-effects models to explore the effect of brain regional cMD at baseline on the longitudinal evolution of tau burden and cognitive performance. Models were corrected for covariates age and sex.

Effect(s) tested: No tasks were used. The study only used structural (not functional) MRI.

Specify type of analysis: ☐ Whole brain ☒ ROI-based ☐ Both

Anatomical location(s): We focused on regional cortical mean diffusivity assessed in two ROIs: the entorhinal and inferior temporal cortices. These regions were used as a proxies for age-related and AD-related tau deposition, respectively.

Statistic type for inference
(See Eklund et al. 2016)

NA. Statistical analyses were performed for specific ROIs only.

Correction

ROI-based statistical analyses were corrected for multiple comparisons using the Benjamini-Hochberg method.

Models & analysis

n/a	Involved in the study
☒	☐ Functional and/or effective connectivity
☒	☐ Graph analysis
☐	☒ Multivariate modeling or predictive analysis

Multivariate modeling and predictive analysis

MODEL 1:
Tau EC or IT ~ Age + Sex + Baseline Tau EC or IT + Baseline cMD EC or IT x Baseline Amyloid Group x Time + (1|Subject)
MODEL 2:
LMDR ~ Age + Sex + Baseline LMDR + Baseline cMD EC or IT x Baseline Amyloid Group x Time + (1|Subject)
